# Hepatoprotective Effects of Polysaccharides Isolated from *Phellinus gilvus* Against Carbon Tetrachloride-induced Liver Injury in Rats

**DOI:** 10.5487/TR.2009.25.1.029

**Published:** 2009-03-01

**Authors:** Seung-Chun Park, Yong-Pil Cheon, Wha-Young Son, Man-Hee Rhee, Tae-Wan Kim, Jae-Chan Song, Kil-Soo Kim

**Affiliations:** 15 Department of Veterinary Toxicology, College of Veterinary Medicine, Kyungpook National University, 1370 Sangyeok-dong, Buk-gu, Daegu, 702-701 Korea; 25 College of Natural Sciences, Sungshin Women’s University, Seoul, 136-742 Korea; 35 College of Veterinary Medicine, Chungnam National University, Daejeon, 305-361 Korea

**Keywords:** *Phellinus gilvus* polysaccharide, Carbon tetrachloride, Hepatoprotective effect

## Abstract

*Phellinus gilvus* (PG) is a widely used mushroom for health promotion. We studied the hepatoprotective effect of the polysaccharide aqueous extract of PG (PGP) against carbon tetrachloride (CCl_4_)-induced liver injury in rats. Sprague Dawley rats were divided into 5 groups: Normal control, CCl_4_ control, PGP 50, 100, and 200 mg/kg + CCl_4_. The levels of serum biochemical parameters, liver lipid peroxide and antioxidant enzymes, and histological appearances were evaluated. The CCl_4_-induced increments of alanine aminotransferase, asparate aminotransferase, and alkaline phosphatase levels in serum were significantly decreased by PGP-pretreatments. The PGP dose-dependently decreased hepatic malondialdehyde formations in CCl_4_-treatment groups. Hepatic antioxidant enzymes (superoxide dismutase, catalase, and glutathione peroxidase) were elevated by PGP in CCl_4_-treatment groups. Histopathological evaluation of liver showed that the loss of hepatocytes, fatty changes, swelling and extensive necrosis of hepatocytes in centrilobular regions of the CCl_4_-treated rats were ameliorated by PGP pretreatment. The PGP has hepatoprotective and antioxidative effects in CCl_4_-induced liver injury of rat.

## INTRODUCTION

Mushrooms have been valued as an important edible and medical resource. A number of bioactive materials have been identified in many mushroom species. Hot water soluble fractions of mushrooms, such as polysaccharides, were mainly used as traditional medicinal materials in East Asia, where the practical usage of mushrooms primarily originated (Wasser, 2002).

*Phellinus gilvus* (PG), *Phellinus linteus* (PL), and *Phellinus baumi* (PB) are mushrooms belonging to the Hymenochaetaceae basidiomycetes (Dai and Xu, 1998). PL and PB are well known as sources of antitumor or immunopotentiating polysaccharides, and they have been used as folk medicines for a variety of human diseases in several Asian countries for a long time. Some researchers reported that PL possess effects of antitumor (Lee *et al.*, 2005), hepatoprotective (Kim *et al.*, 2004), antibacterial (Hur *et al.*, 2004), and antiangiogenic and antioxidant activity (Song *et al.*, 2003). PG or PB could inhibit the increase of neutrophils and the IL-1$\beta$ level of bronchial larvage fluid in lipopolysaccharide-challenged rats, and might be useful in preventing acute pulmonary inflammation in human diseases (Jang *et al.*, 2004). It was also reported that PG had significant reducing effects of adhesion and abscess in a rat peritonitis model, and dermal wound healing effects (Bae *et al.*, 2005a, b). In addition, the capability of PB as an antioxidant, free radical scavenger (Shon *et al.*, 2003) and antidiabetes (Hwang *et al.*, 2005) has recently been reported. Among *Phellinus spp.*, PG has a very short growth period (3 months) compared with PL and PB. Though fruiting bodies of PG have been widely used as health promoting food, no scientific data is available about the protective effects of the polysaccharide aqueous extract of PG (PGP) against liver injury.

The liver is the most important organ concerned with the biochemical activities and has a great capacity to detoxicate harmful substances. There is a growing focus to evaluate the scientific basis for the traditional herbal medicines which are claimed to possess hepatoprotective activity.

Carbon tetrachloride ($CCl_4$) is a xenobiotic which produces hepatotoxicity in humans as well as in animals (Brattin *et al.*, 1985). $CCl_4$ is widely used in animal models to induce acute liver injury. Trichloromethyl radical, which is formed in the metabolism of $CCl_4$ via the liver microsomal cytochrome P450 system, reacts rapidly with molecular oxygen to produce the trichloromethylperoxy radical. These radicals react with unsaturated fatty acids of phospholipids in the cell membrane, initiating lipid peroxidation in the liver cell (Recknagel *et al.*, 1989).

In this study, we examined the hepatoprotective effect of PGP pretreatment prior to $CCl_4$ induced acute liver injury in the rat.

## MATERIALS AND METHODS

Fifty male Sprague Dawley rats (6 weeks old) with a mean body weight of 200~230 g were used (Orient Bio, Korea). All rats were given free access to standard rodent chow(laboratory chow 5057, Purina Korea, Korea) and filtered tap water, and were housed in air-controlled room under standard conditions (temperature; 20 ± 2°C, relative humidity; 50 ± 10%, and 12 hour light/dark cycle). All animal experiments were performed in accordance with the Guidelines for Animal Care and Use Committee of Kyungpook National University. Rats were randomly assigned into 5 groups (n = 10): Group 1 (Normal control; saline alone), Group 2 ($CCl_4$ control; saline + $CCl_4$), Group 3 (PGP 50 mg/kg + $CCl_4$), Group 4 (PGP 100 mg/kg + $CCl_4$), and Group 5 (PGP 200 mg/kg + $CCl_4$).

***Preparation of Polysaccharides isolated from Phellinus gilvus.*** The fruiting body of PG was provided by Gyeongbuk Agriculture Technology Administration (Daegu, Korea). The fruiting bodies of PG were cut into small pieces and dried at 40~50°C for 48 hrs. They were homogenized, extracted under optimal water extraction conditions of DW (1 : 25) at 100°C for 10 hrs, and the aqueous phase was concentrated at 80°C in a rotary evaporator (Büchi Rotavapor R-114, Switzerland) to reduce it to one-tenth of its volume. It was then mixed with 95% ethanol (1 : 3), the precipitate was dialyzed (1 : 100,000) in water, then filtered, centrifuged, redissolved and lyophilized successively to get polysaccharides. PGP was stored at 4°C until used.

***Experimental protocols.*** PGP was diluted with saline and given orally by gavage for 14 days, at daily doses of 50, 100, 200 mg/kg. The $CCl_4$ control group received equal amounts of saline given orally for 14 consecutive days. On the 15th day, acute liver injury was induced by single intraperitoneal injection of 1 ml $CCl_4$ [1 : 1 (v/v) solution of $CCl_4$ and olive oil] per rat (100 g) at 1 hr after the last PGP or saline dosing. The normal control group was injected with an equal volume of saline. Animals from each group were sacrificed under ether anesthesia 24 hrs after $CCl_4$ treatment, blood was withdrawn from the abdominal aorta, and the centrifuged serum was kept at -60°C until analyses. Also, liver tissues were obtained from the rats for determination of malondialdehyde (MDA) and antioxidant enzymes and histopathological examination. The liver was excised, and microsomal fractions were prepared by centrifugation and stored in small aliquots at -80°C until analysis could be performed.

The serum levels of asparate aminotransferase (AST), alanine aminotransferase (ALT), and alkaline phosphatase (ALP) were determined by an automatic clinical analyzer (Hemagen Benchtop Chemistry System, Waltham, USA).

The liver was perfused with 0.9% ice-cold saline to completely remove the blood. Then it was suspended in 10% (w/v) ice-cold phosphate buffer (0.1 M, pH 7.4) cut into small pieces, and weighed and homogenized using a Teflon homogenizer. Liver homogenates were used for the assay of lipid peroxide (Ohkawa *et al.*, 1979), Superoxide dismutase (SOD) (Chan, 1996), Catalase (CAT) (Sinha, 1972), and Glutathione peroxidase (GSH) (Moron *et al.*, 1979). The degree of lipid peroxidation in the liver tissue homogenates of all rats was determined MDA formation. The protein concentration was determined by the method of Lowry *et al.* (1951) using crystalline bovine serum albumin as a standard.

Liver tissues were collected from the same lobe and fixed in 10% neutral buffered formalin solution. Liver tissue was dehydrated and embedded in paraffin. Sections were stained using haematoxylin and eosin for light microscopic observations.

The results were expressed as mean ± SEM. The difference between the means of multiple groups was evaluated with an one-way ANOVA and Tukey's multiple comparison test. If any significance was observed between the groups, Dunnett's test was used to compare the means of 2 specific groups; $P < 0.05$ was considered to be significant.

## RESULTS

The changes of serum ALT, AST and ALP levels are shown in Fig. 1. Serum ALT, AST and ALP levels were

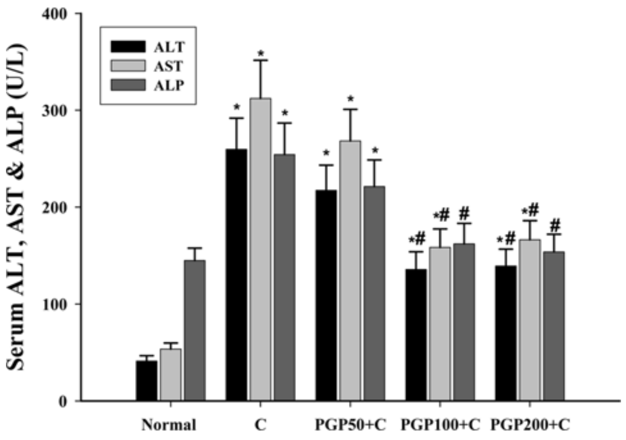

**Fig. 1.** Effect of PGP pretreatment on serum ALT, AST and ALP activities on $CCl_4$-treated rats. C, $CCl_4$ treatment * $P < 0.05$ versus normal control; # $P < 0.05$ versus $CCl_4$ treatment.

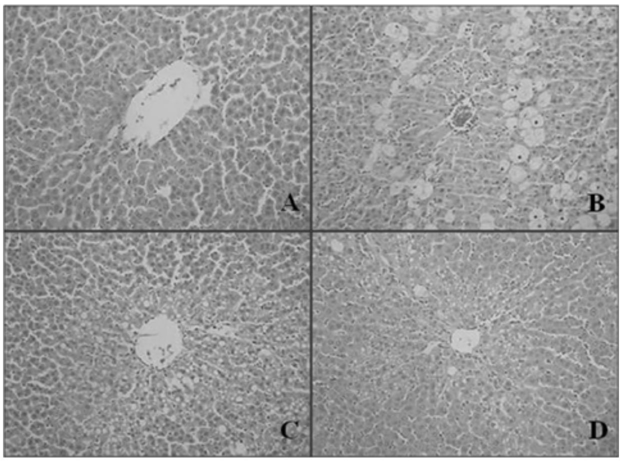

**Fig. 2.** Effects of PGP pretreatment on histopathology of the rat liver after 24 hrs of $CCl_4$-treatment. A, normal group, a little histological change was observed; B, $CCl_4$ control group, loss of hepatocytes, fatty changes, swelling and extensive necrosis of hepatocytes in centrilobular regions were observed; C and D, $CCl_4$-induced liver injury group with PGP pretreatment at different doses of 100 and 200 mg/kg, respectively, reduction in the fatty degeneration, inhibition of hepatic cells necrosis and normalization of fatty change, some inflammation and necrosis were seen (H-E, × 200).

markedly elevated compared with the normal control group at 24 hrs after $CCl_4$ administration. In contrast, the levels were significantly decreased by PGP pretreatment (200 mg/kg) to a maximum of 53.7%, 53.3% and 60.4%, respectively. PGP administration of 100 and 200 mg/kg showed a significant ($p < 0.05$) reduction in the serum ALT, AST and ALP activities compared with $CCl_4$ control group. The protective effect was found to be the highest when the PGP was administered at a dose of 100~200 mg/kg prior to $CCl_4$ administration.

Lipid peroxide (MDA) and antioxidant enzymes (SOD, CAT, and GSH) levels in the liver homogenates of all experimental rats are shown in Table 1. Liver MDA contents were significantly increased to 1.673 μmol/mg protein in rats treated with $CCl_4$ compared with the normal control. Pretreatments of PGP at the doses of 50, 100 and 200 mg/kg were dose-dependently decreased the levels of MDA at 72 hrs after $CCl_4$ administration to 0.965, 0.931 and 0.847 μmol/mg protein, respectively.

**Table 1.** Effect of PGP pretreatment on levels of hepatic lipid peroxide and antioxidant enzymes in $CCl_4$-treated rats

| Groups | Hours | MDA (μmol/mg protein) | SOD (U/mg protein) | GSH (μg/mg protein) | CAT (μmol/mg protein) |
|---|---|---|---|---|---|
| Normal | 24 | 0.598 ± 0.039 | 5.93 ± 0.76 | 9.22 ± 0.61 | 67.33 ± 5.46 |
| | 48 | 0.561 ± 0.017 | 5.57 ± 0.91 | 9.35 ± 0.66 | 65.17 ± 4.74 |
| | 72 | 0.587 ± 0.025 | 5.49 ± 0.84 | 9.39 ± 0.96 | 69.14 ± 5.27 |
| $CCl_4$ | 24 | 1.673 ± 0.097* | 2.65 ± 0.63* | 5.36 ± 0.47* | 48.71 ± 4.01* |
| | 48 | 1.421 ± 0.073* | 2.52 ± 0.67* | 5.14 ± 0.43* | 45.02 ± 4.14* |
| | 72 | 1.074 ± 0.081* | 3.02 ± 0.58* | 5.96 ± 0.39* | 52.97 ± 4.67* |
| $CCl_4$ + PGP50 | 24 | 1.488 ± 0.092*# | 2.78 ± 0.47* | 5.63 ± 0.56* | 51.67 ± 4.38* |
| | 48 | 1.126 ± 0.074*# | 2.96 ± 0.58* | 5.58 ± 0.51* | 54.13 ± 4.01*# |
| | 72 | 0.965 ± 0.061* | 3.03 ± 0.66* | 5.97 ± 0.54* | 59.85 ± 6.62 |
| $CCl_4$ + PGP100 | 24 | 1.421 ± 0.088* | 3.82 ± 0.58* | 6.36 ± 0.69* | 54.36 ± 4.61* |
| | 48 | 1.201 ± 0.072*# | 4.11 ± 0.51# | 6.81 ± 0.62*# | 59.32 ± 3.58# |
| | 72 | 0.931 ± 0.058*# | 4.39 ± 0.46# | 7.56 ± 0.73# | 62.01 ± 4.03# |
| $CCl_4$ + PGP200 | 24 | 1.032 ± 0.074*# | 4.02 ± 0.39*# | 6.49 ± 0.62* | 59.16 ± 4.77*# |
| | 48 | 0.961 ± 0.080*# | 4.96 ± 0.28# | 6.68 ± 0.61*# | 61.25 ± 4.61# |
| | 72 | 0.847 ± 0.069*# | 4.87 ± 0.40# | 7.72 ± 0.74# | 62.18 ± 4.81# |

Each value is the mean ± SEM.
MDA: malondialdehyde; SOD: superoxide dismutase; CAT: catalase; GSH: glutathione peroxidase.
*$P < 0.05$ versus normal control; #$P < 0.05$ versus $CCl_4$ control.

Hepatic SOD, CAT, and GSH levels were significantly decreased after $CCl_4$ treatment in comparison with the normal control. PGP pretreatment (100 and 200 mg/kg) remarkably increased SOD, CAT, and GSH activities in liver.

The liver sections in PGP pretreated rats and normal control rats at 24 hrs after $CCl_4$-treatment are shown in Fig. 2. Liver tissue section of normal control is shown in Fig. 2A. In $CCl_4$ control group, the histology at 24 hrs after $CCl_4$ administration showed loss of hepatocytes, fatty changes, swelling and extensive necrosis of hepatocytes in centrilobular regions (Fig. 2B). In contrast, PGP pretreatment (100 mg/kg) showed less severe lobular damage of hepatocytes compared with $CCl_4$ control group (Fig. 2C). Together with hepatocytes in the centrilobular area of the $CCl_4$-treated group, PGP pretreatment (200 mg/kg) showed fewer necrotic and degenerative changes and little inflammatory cell infiltration by PGP dose dependent manner (Fig. 2D).

## DISCUSSION

The present study was conducted to investigate whether polysaccharides isolated from PG could inhibit $CCl_4$ induced hepatic injury in rats in order to assess the scientific and therapeutic validation of PG.

PGP pretreatment (100 and 200 mg/kg) significantly inhibited the elevation of ALT, AST, and ALP levels by $CCl_4$ (Fig. 1). These results suggest that PGP has liver cell regenerative effects and is able to stabilize membrane structures in hepatic injury. However, under the present experimental single dose (1 ml/kg body weight), there were no changes in the other serum biochemical parameters at 24 hrs after $CCl_4$ administration.

MDA, a stable metabolite of the free radical-mediated lipid peroxidation cascade, is widely used as a marker of lipid peroxidation (Mansour, 2000). The level of MDA formation is an indirect measurement of lipid peroxidation. Lipid peroxide levels in liver tissue were found to be significantly elevated in $CCl_4$-treated rats. It has been shown that enhanced lipid peroxidation occurs with the development of injury in the liver of rats treated with $CCl_4$. PGP pretreatment decreased the elevated level of hepatic MDA by 20% at 24 hrs after $CCl_4$ administration. Furthermore, PGP (100 and 200 mg/kg) pretreated group showed almost normal MDA formation in liver. Our results indicated that PGP possesses potent antioxidative activity and protects liver against $CCl_4$-induced oxidative stress and hepatotoxicity.

PGP pretreatment remarkably increased SOD, CAT, and GSH activities in liver in comparison with the $CCl_4$ control. Cells have a number of mechanisms to protect from the toxic effects of $CCl_4$ including free radical scavengers and chain reaction terminators such as SOD, CAT, and GSH systems. These results indicate that PGP is effectively able to inhibit production of MDA and potentiate antioxidative activity in $CCl_4$-injured liver.

PGP pretreatment ameliorates $CCl_4$-induced hepatic pathological changes such as loss of hepatocytes, fatty changes, swelling, cytoplasmic vacuolization and necrosis of hepatocytes in centrilobular regions of the liver. Especially, PGP pretreatment (200 mg/kg) reduced the fatty degeneration and hepatic cells necrosis and showed almost normalization. Therefore, although the mechanism of action may not be identified, PGP appears to exert a protective effect against $CCl_4$-induced hepatic injury.

The results of the present study demonstrated that pretreatment with PGP had potent hepatoprotective and hepatic regenerative effects against $CCl_4$-induced hepatotoxicity in rats, as evidenced by serum ALT, AST, and ALP activities, hepatic lipid peroxide and antioxidant enzymes (SOD, CAT, and GSH) levels, and histopathological appearances of liver. Moreover, these protective effects were found to be dose-dependent. Therefore, the PGP may give great health benefits to humans and animals with hepatic injuries. Further works are needed to fully characterize PGP and elucidate its possible mode of action and this work is in progress.

## ACKNOWLEDGEMENTS

This work was supported by a grant (ARPC 105036-3) of the Technology Development Program for agriculture and Forestry, Ministry of Agriculture and Forestry, Republic of Korea.
